# A Reverse Transcription Loop-Mediated Isothermal Amplification Assay Optimized to Detect Multiple HIV Subtypes

**DOI:** 10.1371/journal.pone.0117852

**Published:** 2015-02-12

**Authors:** Karen E. Ocwieja, Scott Sherrill-Mix, Changchun Liu, Jinzhao Song, Haim Bau, Frederic D. Bushman

**Affiliations:** 1 Department of Microbiology, Perelman School of Medicine at the University of Pennsylvania, Philadelphia, Pennsylvania, United States of America; 2 Department of Mechanical Engineering and Applied Mechanics, School of Engineering and Applied Science, University of Pennsylvania, Philadelphia, Pennsylvania, United States of America; University of Brighton, UNITED KINGDOM

## Abstract

Diagnostic methods for detecting and quantifying HIV RNA have been improving, but efficient methods for point-of-care analysis are still needed, particularly for applications in resource-limited settings. Detection based on reverse-transcription loop-mediated isothermal amplification (RT-LAMP) is particularly useful for this, because when combined with fluorescence-based DNA detection, RT-LAMP can be implemented with minimal equipment and expense. Assays have been developed to detect HIV RNA with RT-LAMP, but existing methods detect only a limited subset of HIV subtypes. Here we report a bioinformatic study to develop optimized primers, followed by empirical testing of 44 new primer designs. One primer set (ACeIN-26), targeting the HIV integrase coding region, consistently detected subtypes A, B, C, D, and G. The assay was sensitive to at least 5000 copies per reaction for subtypes A, B, C, D, and G, with Z-factors of above 0.69 (detection of the minor subtype F was found to be unreliable). There are already rapid and efficient assays available for detecting HIV infection in a binary yes/no format, but the rapid RT-LAMP assay described here has additional uses, including 1) tracking response to medication by comparing longitudinal values for a subject, 2) detecting of infection in neonates unimpeded by the presence of maternal antibody, and 3) detecting infection prior to seroconversion.

## Introduction

Despite the introduction of efficient antiretroviral therapy, HIV infection and AIDS continue to cause a worldwide health crisis [1]. Methods for detecting HIV infection have improved greatly with time [2]—today rapid assays are available that can detect HIV infection in a yes-no format using a home test kit that detects antibodies in saliva. Viral load assays that quantify viral RNA with quick turn-around time are widely available in the developed world. However, quantitative viral load assays are not commonly available with actionable time scales in much of the developing world. This motivates the development of new rapid and quantitative assays that can be used at the point of care with minimal infrastructure [3,4].

One simple and quantitative detection method involves reverse transcription-based loop mediated isothermal amplification (RT-LAMP) [5]. In this method, a DNA copy of the viral RNA is generated by reverse transcriptase, and then isothermal amplification is carried out to increase the amount of total DNA. Primer binding sites are chosen so that a series of strand displacement steps allow continuous synthesis of DNA without requiring thermocycling. Reaction products can be detected by adding an intercalating dye to reaction mixtures that fluoresces only when bound to DNA, allowing quantification of product formation by measurement of fluorescence intensity. Such assays can potentially be packaged in simple self-contained devises and read out with no technology beyond a cell phone.

RT-LAMP assays for HIV-1 have been developed previously and reported to show high sensitivity and specificity for subtype B, the most common HIV strain in the developed world [4,6,7]. Another recent study reported RT-LAMP primer set optimized for the detection of HIV variants circulating in China [13], and another on confirmatory RT-LAMP for group M viruses [14]. Assays have also been developed for HIV-2 [8]. A complication arises in using available RT-LAMP assays due to the variation of HIV genomic sequences among the HIV subtypes [9,10], so that an RT-LAMP assay optimized for one viral subtype may not detect viral RNA of another subtype [11]. Tests presented below show that many RT-LAMP assays are efficient for detecting subtype B, for which they were designed, but often performed poorly on other subtypes. Subtype C infects the greatest number of people worldwide, including in Sub-Saharan Africa, where such RT-LAMP assays would be most valuable, motivating optimization for subtype C. Several additional non-B subtypes are also responsible for significant burdens of disease world-wide [12].

Here we present the development of an RT-LAMP assay capable of detecting HIV-1 subtypes A, B, C, D, and G. We first carried out a bioinformatic analysis to identify regions conserved in all the HIV subtypes. We then tested 44 different combinations of RT-LAMP primers targeting this region in over 700 individual assays, allowing identification of a primer set (ACeIN-26) that was suitable for detecting these subtypes. We propose that the optimized RT-LAMP assay may be useful for quantifying HIV RNA copy numbers in point-of-care applications in the developing world, where multiple different subtypes may be encountered.

## Results

### Testing published RT-LAMP primer sets against multiple HIV subtypes

We first assessed the performance of existing RT-LAMP assays on RNA samples from multiple HIV subtypes. We obtained viral stocks from HIV subtypes A, B, C, D, F, and G, estimated the numbers of virions per ml, and extracted RNA. RNAs were mixed with RT-LAMP reagents which included the six RT-LAMP primers, designated F3, B3, FIP, BIP, LF and LB [5]. Reactions also contained reverse transcriptase, DNA polymerase, nucleotides and the intercalating fluorescent EvaGreen dye, which yields a fluorescent signal upon DNA binding. DNA synthesis was quantified as the increase in fluorescence intensity over time, which yielded a typical curve describing exponential growth with saturation (examples are shown below). Results are expressed as threshold times (T_t_) for achieving 10% of maximum fluorescence intensity at the HIV RNA template copy number tested.

**Fig 1:**
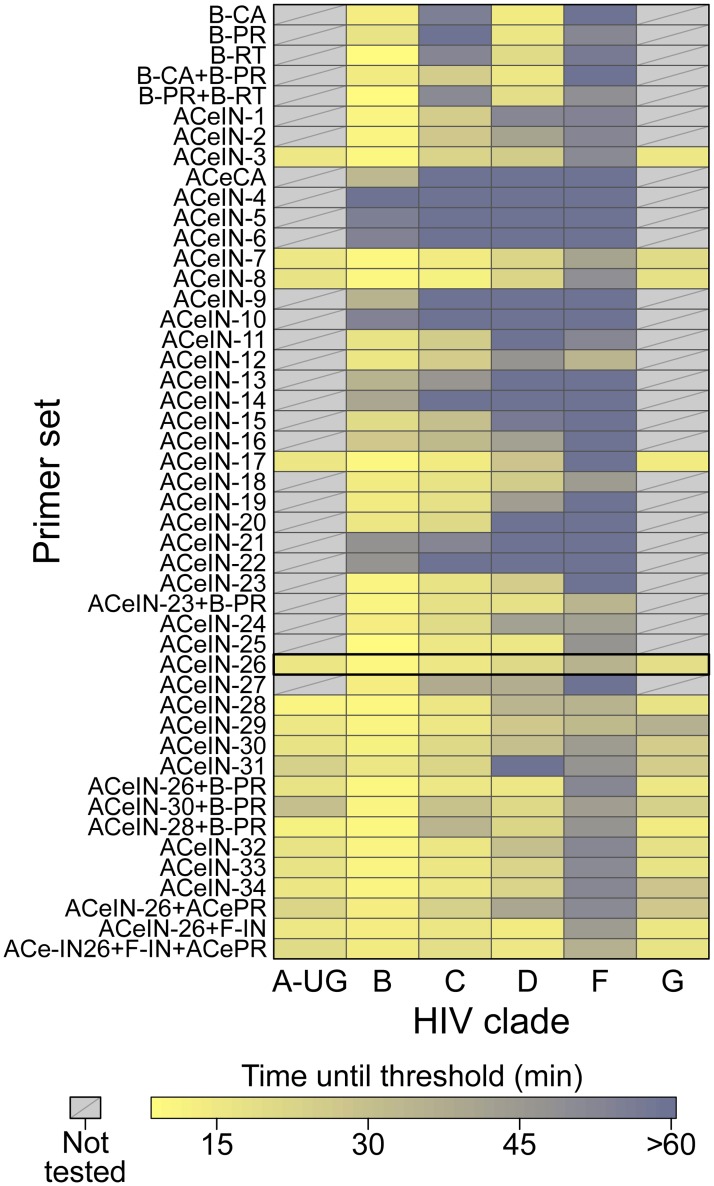
Summary of amplification results for all the RT-LAMP primer sets tested in this study. The data is shown as a heat map, with more intense yellow coloring indicating shorter amplification times (key at bottom). Primer sets tested are named along the left of the figure. Primer sequences, and their organization into LAMP primer sets, are cataloged in S1 and S2 Tables. The raw data and averaged data are collected in S3 and S4 Tables. ACeIN-26 primer set (highlighted) had one of the best performances across the subtypes and a relatively simple primer design.

In initial tests, published primer sets targeting the HIV-1 subtype B coding regions for capsid (CA), protease (PR), and reverse transcriptase (RT) (named B-CA, B-PR and B-RT) were assayed in reactions with RNAs from four of the subtypes. Results with each primer set tested are shown in Fig. 1 in heat map format, where each tile summarizes the results of tests of 5000 RNA copies. Primers and their groupings into sets are summarized in S1 and S2 Tables, average assay results are in S3 Table, and raw assay data is in S4 Table. Assays (Fig. 1, top) with the B-CA, B-PR and B-RT primer sets detected subtypes B and D at 5000 RNA copies with threshold times less than 20 min. However, assays with B-CA and B-RT detected subtypes C and F with threshold times >50 min, indicating inefficient amplification and the potential for poor separation between signal and noise. B-PR did not detect subtype C at all. In an effort to improve the breadth of detection, we first tried mixing the B-PR primers, which detected clade F (albeit with limited efficiency) with the B-CA and B-RT primers (Fig. 1 and S3 and S4 Tables). In neither case did this provide coverage of all four clades tested. We thus did not test these primer sets on RNAs from the remaining subtypes and instead sought to develop primer sets targeting different regions of the HIV genome.

### Primer design strategy

**Fig 2:**
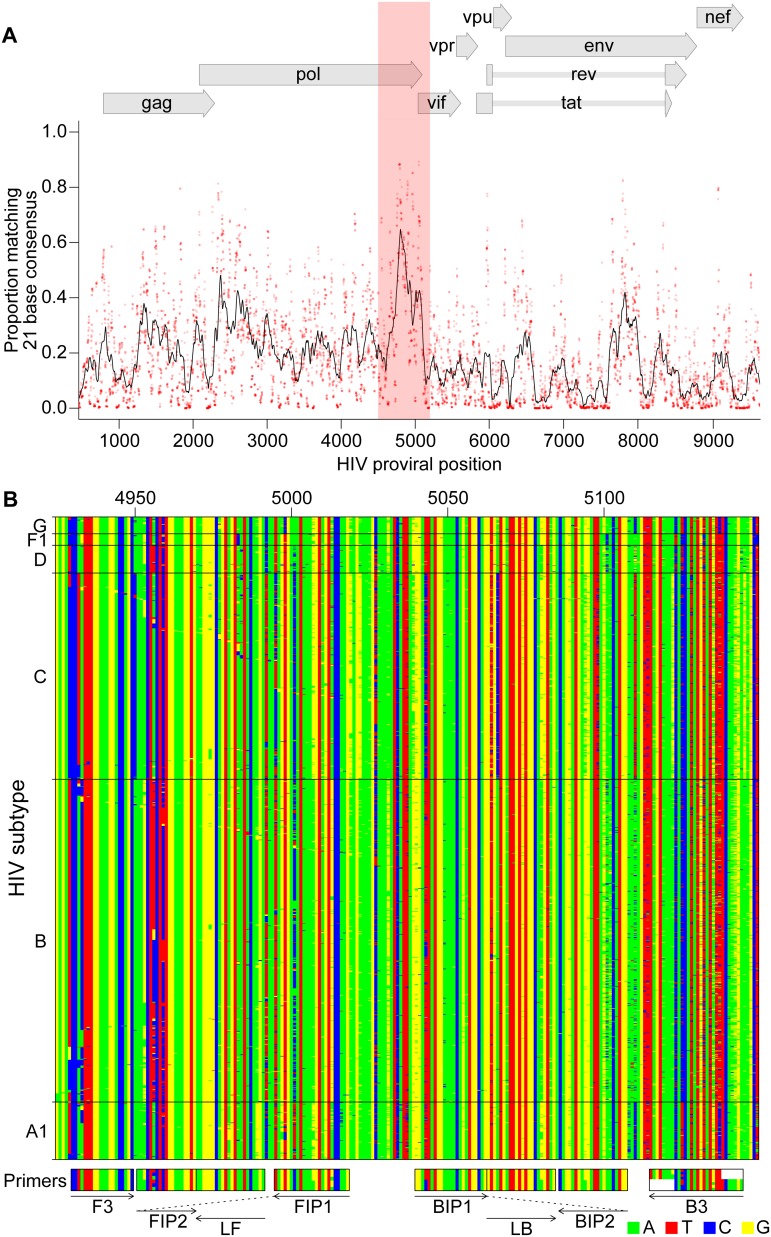
Bioinformatic analysis to design subtype-agnostic RT-LAMP primers. A) Conservation of sequence in HIV. HIV genomes (n = 1340) from the Los Alamos National Laboratory collection (file HIV1_ALL_2012_genome_DNA.fasta; http://www.hiv.lanl.gov/content/sequence/NEWALIGN/align.html#web) were aligned and conservation calculated. The x-axis shows the coordinate on the HIV genome, the y-axis shows the proportion of sequences matching the consensus for each 21 base segment of the genome (red points). The black line shows a 101 base sliding average over these proportions. The vertical red shading shows the region targeted for LAMP primer design that was used as input into the EIKEN primer design tool. Numbering is relative to the HIV_89.6_ sequence. B) Aligned genomes, showing the locations of the ACeIN-26 primers. Sequences in the red shaded region in A are shown, with DNA bases color-coded as shown at the lower right. Each row indicates an HIV sequence and each column a base in that sequence. Horizontal lines separate the HIV subtypes (labeled at right). Arrows indicate the strand targeted by each primer. Primers targeting the negative strand of the virus are shown as reverse compliments for ease of viewing.

To design primers that detected multiple HIV subtypes efficiently, we analyzed alignments of HIV genomes (downloaded from the Los Alamos National Laboratory site [9]) for regions with similarity across most viruses, revealing that a segment of the pol gene encoding IN was particularly conserved (Fig. 2A). A total of six primers are required for each RT-LAMP assay [5]. We used the EIKEN primer design tool to identify an initial primer set targeting this region. In further analysis, positions in the alignments were identified within primer landing sites that commonly contained multiple different bases. Primer positions were manually adjusted to avoid these bases when possible, and when necessary mixtures were formulated containing each of these commonly occurring bases (S1 and S2 Tables). An extensive series of variants targeting the IN coding region was tested empirically in assays containing RNAs from multiple subtypes (5000 RNA copies per reaction, over 700 total assays; S3 and S4 Tables). Based on initial results, primers were further modified by adjusting the primer position or addition of locked nucleic acids as described below.

### Testing different primer designs

Our first design, ACeIN-1 (“ACe” for “All Clade”, and “IN” for “integrase”), targeted the HIV IN coding region and contained multiple bases at selected sites to broaden detection (Fig. 1). ACeIN-2 and-3 have primers (B3) with slightly different landing sites. Tests showed that the mixture of primers allowed amplification with a shorter threshold time than did either alone (Fig. 1).

We also tried to design a new primer set to the CA coding region (Fig. 1, ACeCA) but found that the set only amplified clade B, and not efficiently. Thus this design was abandoned.

ACeIN-3 through-6 were altered by inserting a polyT sequence between the two different sections of FIP and BIP in various combinations, a modification introduced with the goal of improving primer folding, but these designs performed quite poorly (Fig. 1).

Because the FIP primer appeared to bind the region with most variability among clades, we tried variations that bound to several nearby regions. These were tried with and without the polyT containing BIP and FIP primers in various combinations (Fig. 1, ACeIN-7 through-22). We also tried mixing all of the variations of FIP together (ACeIN-23; S2 Table). The ACeIN-23 primer set was tried as a mixture with the B-PR set to try to capture clade F, yielding a relatively effective primer set (Fig. 1, ACeIN-23+B-PR).

In an effort to increase affinity, an additional G/C pair was added to F3 and tested with various other IN primers (Fig. 1, ACeIN-24 through-31). Testing showed improvement, with ACeIN-26 showing particularly robust amplification.

In a second effort to increase primer affinities, we substituted locked nucleic acids (LNAs) for selected bases that were particularly highly conserved among subtypes (Fig. 1, ACeIN-30, -31, -32, -33, and-34). Some improvement was shown over the non-LNA containing bases. However, the ACeIN-26 primer set was as effective as or better than any LNA containing primer sets.

In further tests, the ACeIN-26, -28 and-30 primers were tested combined with the ACePR primer set (a slightly modified version of the B-PR primer set, S2 Table, row 2, designed to accommodate a wider selection of HIV-1 subtypes) but no improvement was seen and efficiency may even have fallen for some subtypes. We also designed a primer set that matched exactly to the targeted sequences found in the problematic subtype F, and mixed this set with the ACeIN-26 primers. However, no improvement was seen (Fig. 1, mixtures with F-IN set). Mixing the ACeIN-26 primers with both the ACePR and F-specific primers did yield effective primer sets (Fig. 1, ACeIN26+F-IN and ACeIN26+F-IN+ACePR). However, amplification efficiency was not greatly improved over the ACeIN-26 primer set, so we proceeded with the simpler ACeIN-26 primer set in further studies.

### Performance of the optimized RT-LAMP assay

**Fig 3:**
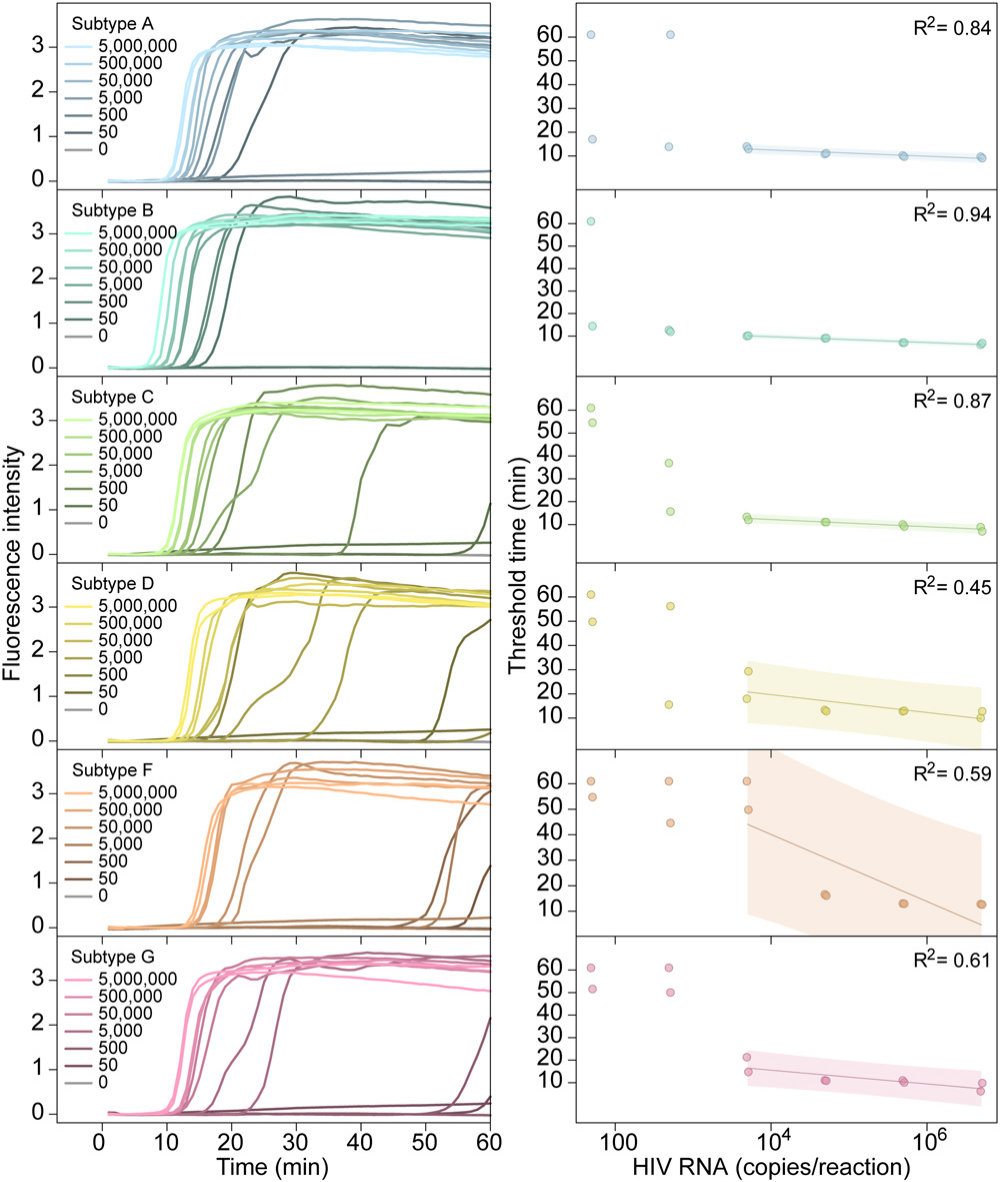
Performance of the AceIN-26 primer set with different starting RNA concentrations. Tests of each subtype are shown as rows. In each lettered panel, the left shows the raw accumulation of fluorescence signal (y-axis) as a function of time (x-axis); the right panel shows the threshold time (y-axis) as a function of log RNA copy number (x-axis) added to the reaction. In the right hand panels, values were dithered where two points overlapped to allow visualization of both.

The ACeIN-26 RT-LAMP primer set was next tested to determine the minimum concentration of RNA detectable under the reaction conditions studied (Fig. 3). RNA template amounts were titrated and time to detection quantified. Tests showed detection after less than 20 min of incubation for 50 copies of subtypes A or B, detection after less than 30 min for 5000 copies for C, D, and G, and detection after less than 20 min for 50,000 copies for F.

**Fig 4:**
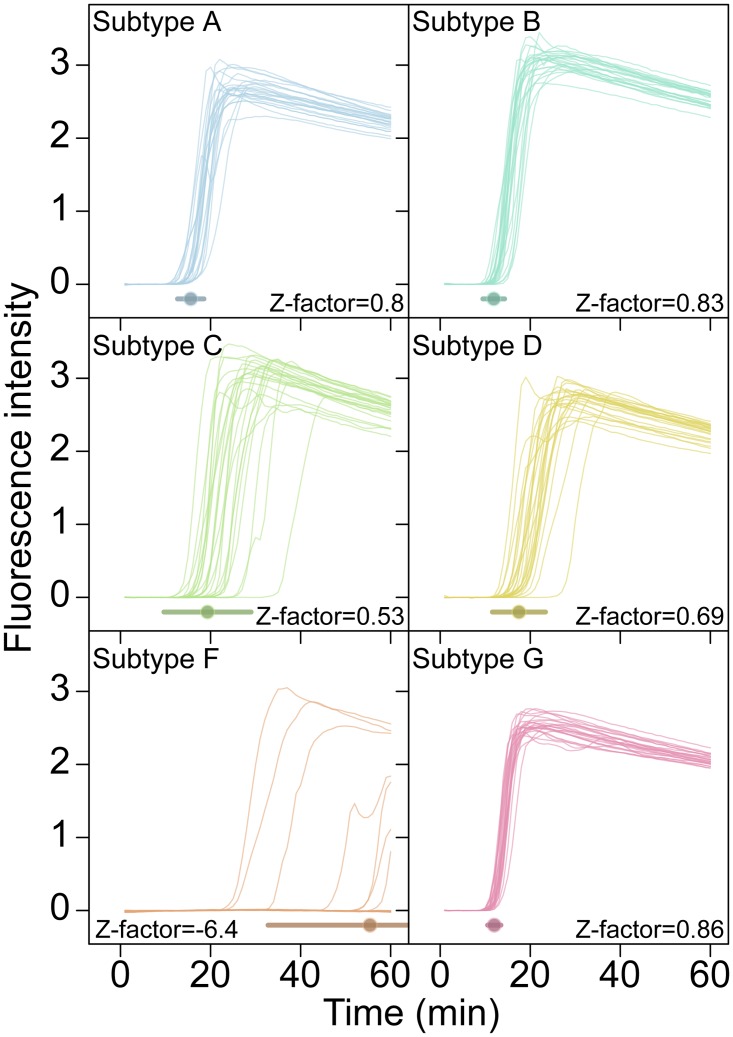
Examples of time course assays, displaying replicate tests of RT-LAMP primer set ACeIN-26 tested over six HIV subtypes, used in Z-factor calculations. A total of 5000 RNA copies were tested in each 15 μL reaction. Time is shown on the x-axis, Fluorescence intensity on the y-axis. Replicates are distinguished using an arbitrary color code. Z-factor values and standard deviations are shown on each panel.

For clinical implementation the reliability of an assay is critical. This is commonly summarized as a Z-factor [15], which takes into account both the separation in means between positive and negative samples and the variance in measurement of each. An assay with a Z-factor above 0.5 is judged to be an excellent assay. Z-factors for detection of each of the subtypes at 5000 RNA copies per reaction were >0.50 for subtypes A, B, C, D, and G, respectively (Fig. 4, n = 24 replicates per test). Detection of subtype F at 5000 copies per reaction was sporadic, showing a much lower Z-factor. Therefore our ACeIN-26 RT-LAMP primer set appears well suited to detect 5000 copies of subtypes A, B, C, D and G.

## Discussion

Here we present an RT-LAMP assay optimized to identify multiple HIV subtypes. Infections with subtype B predominate in most parts of the developed world, but elsewhere other subtypes are more common [12]. Thus nucleic acid-based assays for use in the developing world need to query HIV subtypes more broadly. Previously reported RT-LAMP assays, while effective at detecting subtype B, commonly showed poor ability to detect at least some of the HIV subtypes, including C, which is common in the developing world (Fig. 1). Here we first carried out an initial bioinformatic survey to identify regions conserved across all HIV subtypes that could serve as binding sites for RT-LAMP primers. We then tested primer sets targeting these regions empirically for efficiency. Testing 44 different primer sets revealed that assays containing ACeIN-26 were effective in detecting 5000 copies of RNA from subtypes A, B, C, D, and G within 30 minutes of incubation. For these five subtypes, the times of incubation to reach the threshold times were not too different, which simplifies interpretation when the subtype in the sample is unknown. Regardless of the efficiency, these assays can be applied to longitudinal studies of changes in viral load within an individual. We propose that RT-LAMP assays based on the ACeIN-26 primer set can be useful world-wide for assaying HIV-1 viral loads in infected patients.

There are several limitations to our study. Subtypes A, B, C, D, and G were detected efficiently and showed Z-factors above 0.5, but subtype F was detected reliably only with higher template amounts, probably due to more extensive mismatches with the ACeIN-26 primer set. Subtype F is estimated, however, to comprise only 0.59% of all infections globally [12], though it is common in some regions. For many of the common circulating recombinant forms, such as AE and BC, the target site for ACeIN-26 is from a subtype known to be efficiently detected, though in some cases the efficiency of detection is not easy to predict and will need to be tested. We did not test subtypes beyond A, B, C, D, F and G, and we did not attempt to assess multiple different variants within each subtype. Thus, while we do know that our RT-LAMP assays are more widely applicable than many of those reported previously, we do not know whether they are able to detect all strains efficiently. In addition, although we carried out more than 700 assays in this study, there remain multiple parameters that could be optimized further, such as primer concentrations, salt type and concentration, temperature, and divalent metal concentrations, so there are likely further opportunities for improvement. Also, possible effects of RNA quality on assay performance were not tested rigorously.

A particularly important parameter for further optimization is primer sequence. Several groups have recently published primer sets optimized for broad detection of different HIV lineages [13,14], offering opportunities for creating sophisticated primer blends with increased breadth of detection. However, in developing such mixtures, it will be important to monitor for possible complicating interactions of primers with each other. As an example of ongoing development of mixtures, we found that addition of another primer to the ACeIN-26 set that was matched to a common subtype C lineage allowed improved detection of subtype C variants (S1 Report). In order to improve detection of subtype F, which was suboptimal with ACeIN-26, additional primer sets could be mixed to specifically target subtype F, though the ones we tried so far did not work well. It will be useful to explore the performance of broader primer mixtures in future work.

Today rapid assays are available that can report infection efficiently, for example by detecting anti-HIV antibodies in oral samples—however, the nucleic acid-based method presented here has additional potential uses. We envision combining the RT-LAMP assay with simple point-of-care devices for purifying blood plasma [3] and quantitative analysis of accumulation of fluorescent signals [16]. In one implementation of the technology, cell phones could be used to capture and analyze results, thereby minimizing equipment costs. Point-of-care devices are available facilitating the concentration of viral RNA from blood plasma or saliva [16] to allow the detection of the 1000 RNA copy threshold that the WHO defines as virological treatment failure (World Health Organization, Consolidated ARV guidelines, June 2013). Together, these methods will allow assessment of parameters beyond just the presence/absence of infection. Quantitative RT-LAMP assays should allow tracking of responses to medication, detection in neonates (where immunological tests are confounded by presence of maternal antibody), and early detection before seroconversion.

## Methods

### Viral strains used in this study

Viral strains tested included HIV-1 92/UG/029 (Uganda) (subtype A, NIH AIDS Reagent program reagent number 1650), HIV-1 THRO (subtype B, plasmid derived, University of Pennsylvania CFAR) [17], CH269 (subtype C, plasmid derived, University of Pennsylvania CFAR) [17], UG0242 (subtype D, University of Pennsylvania CFAR), 93BRO20 (subtype F, University of Pennsylvania CFAR), HIV-1 G3 (subtype G, NIH AIDS Reagent program reagent number 3187) [18].

Viral stocks were prepared by transfection and infection. Culture supernatants were cleared of cellular debris by centrifugation at 1500g for 10 min. The supernatant containing virus was then treated with 100 U DNase (Roche) per 450 uL virus for 15 min at 30°C. RNA was isolated using the QiaAmp Viral RNA mini kit (Qiagen GmbH, Hilden, Germany). RNA was eluted in 80 uL of the provided elution buffer and stored at -80°C.

Concentration of viral RNA copies was calculated from p24 capsid antigen capture assay results provided by the University of Pennsylvania CFAR or the NIH AIDS-reagent program. In calculating viral RNA copy numbers, we assumed that all p24 was incorporated in virions, all RNA was recovered completely from stocks, 2 genomes were present per virion, 2000 p24 molecules per viral particle, and the molecular weight of HIV-1 p24 was 25.6 kDa.

### Assays

RT-LAMP reaction mixtures (15 μL) contained 0.2 μM each of primers F3 and B3 (if a primer set used multiple B3 primers, mixture contained 0.2 μM of each); 1.6 μM each of FIP and BIP primers (if a primer set had multiple FIP primes, reaction mixture contained 0.8 μM of each FIP primer); and 0.8 μM each of LoopF and LoopB primers; 7.5 μL OptiGene Isothermal Mastermix ISO-100nd (Optigene, UK), ROX reference dye (0.15 μL from a 50X stock), EvaGreen dye (0.4 μL from a 20X stock; Biotium, Hayward, CA); HIV RNA in 4.7 μL; AMV reverse transcriptase (10U/μL) 0.1 μL; and water to 15 μL. In most cases where two primer sets were combined, the total primer concentration within the reaction was doubled such that the above individual primer molarities were maintained. For the mixture ACeIN-26+F-IN (S2 Table, line 46), the total primer concentration was not doubled—the F-IN primer set comprised 25% of the total primer concentration, and the ACeIN-26 primer set comprised 75% of the total primer concentration with the ratios of primers listed above preserved. This mixture was combined 1:1 with the ACe-PR primer set (S2 Table, line 47) such that total primer concentration in the final mixture was doubled.

Amplification was measured using the 7500-Fast Real Time PCR system from Applied Biosystems with the following settings: 1 minute at 62°C; 60 cycles of 30 seconds at 62°C and 30 seconds at 63°C. Data was collected every minute. Product structure was assessed using dissociation curves which showed denaturation at 83°C. Products from selected amplification reactions were analyzed by agarose gel electrophoresis and showed a ladder of low molecular weight products (data not shown).

Product synthesis was quantified as the cycle of threshold for 10% amplification. Z-factors [15] were calculated from tests of 24 replicates using the ACeIN26 primer set in assays with viral RNA of each subtype. No detection after 60 min was given a value of 61 min in the Z-factor calculation.

## Supporting Information

S1 Report This report describes amplification results obtained with the addition of a BIP primer to the ACeIN-26 primer set to improve detection of subtype C viruses. (DOCX)

S1 Table Primer sequences. This table catalogs all the primer sequences used in this study.
(PDF)

S2 Table HIV RT-LAMP primer sets studied. This table catalogs the primers used in each primer set.
(PDF)

S3 Table Average threshold times. Reactions contained 5000 copies of HIV-1 RNA templates from the subtypes listed at the tops of the columns. The threshold time (T_t_) is defined as the reaction time that elapses until the fluorescent signal increases 10% of maximum fluorescence intensity (I_max_) achieved in RNA-containing reactions.
(PDF)

S4 Table All threshold times generated in this study. This table documents the raw values for results of this study.
(PDF)
