# Supplementary material for: A Reverse Transcription Loop-Mediated Isothermal Amplification Assay Optimized to Detect Multiple HIV Subtypes
Source: PLoS One. 2015 Feb 12;10(2):e0117852. doi: 10.1371/journal.pone.0117852 (PMC4326360; doi:10.1371/journal.pone.0117852)
Supplement: S1 Report — (DOCX) [file pone.0117852.s001.docx]

**Supplementary Report 1**

**This report presents a further modifiction of the ACeIN-26 primer set to improve detection of some subtype C lineages.**

To improve the amplification efficiency of HIV-1 subtype C, we modified the

1. AceIN-BIP primer in ACeIN-26 primer set

GGAYTATGGAAAACAGATGGCAGCCATGTTCTAATCYTCATCCTG

to better match the HIV subtype C sequence. The modified base site in the modified AceIN-BIP primer (ii) is shaded with green (R=A, G) below. The mixed primer consists of a 50% R=A and 50% R=G blend.

1. Modified AceIN-BIP primer

GGAYTATGGAAAACAGATGGCAGCCATGTTCTRATCYTCATCCTG

Fig. S1 evaluates the performance of the modified ACeIN-26 primer set with HIV subtypes B and C. Comparison of Fig. S1-A with the corresponding panel in Fig. 4 of the main text indicates that the modified ACeIN-26 primer set improved reproducibility and led to a higher Z factor (Z factor=0.945, n=7) (**Figure S1 (A)**) than that of ACeIN-26 primer set (Z factor=0.53, n=24) (**Figure 4**) when amplifying HIV subtype C. The modified primer set had no adverse effect on the amplification of HIV subtype B (**Figure S1-B**).


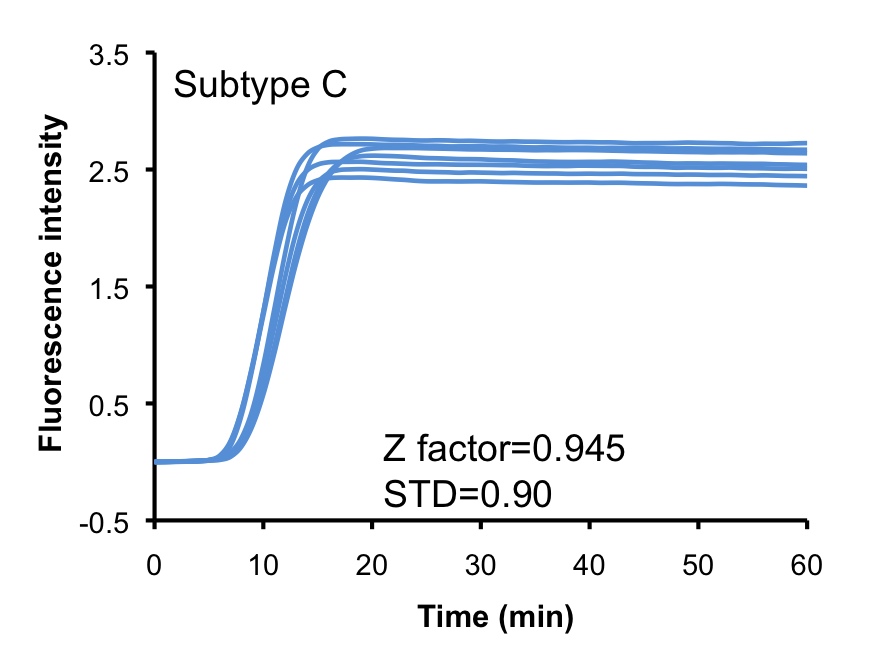


**(A)**

**
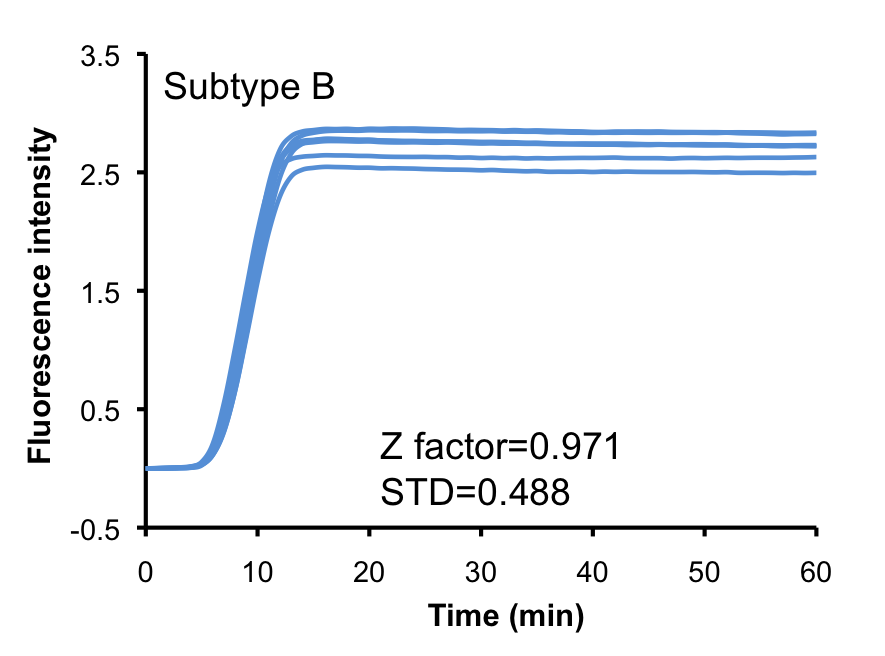
**

**(B)**

**Figure S1:** Emission intensity (arbitrary units) as a function of time when amplifying HIV subtypes C (**A, n=7**) and B (**B, n=7**) with modified ACeIN-26 primer set.
