# Supplementary material for: A Reverse Transcription Loop-Mediated Isothermal Amplification Assay Optimized to Detect Multiple HIV Subtypes
Source: PLoS One. 2015 Feb 12;10(2):e0117852. doi: 10.1371/journal.pone.0117852 (PMC4326360; doi:10.1371/journal.pone.0117852)
Supplement: S1 Table — This table catalogs all the primer sequences used in this study. (PDF) [file pone.0117852.s002.pdf]

Table S1. Primers sequences

Primer	Sources	Sequence (5' to 3')
B-CA-F3	[1]	ATTATCAGAAGGAGCCACC
B-CA-B3	[1]	CATCCTATTTGTTCCTGAAGG
B-CA-FIP	[1]	CAGCTTCCTCATTGATGGTTTCTTTTTAACACCATGCTAAACACAGT
B-CA-BIP	[1]	TGTTGCACCAGGCCAGATAATTTTGTACTGGTAGTTCCTGCTATG
B-CA-LF	[1]	TTTAACATTTGCATGGCTGCTTGAT
B-CA-LB	[1]	GAGATCCAAGGGGAAGTGA
ACeCA-F3	This work	CCCTTTAGAGACTATGTAGACC
ACeCA-B3	This work	TCCTACTCCCTGACATGC
ACeCA-FIP	This work	CAACAAGGTTTCTGTCATCCAATTTGGTTCTATAAAACTCTAAGAGCC
ACeCA-BIP	This work	GTCCAAAATGCGAACCCAGATGTCATCATTTCTTCTAGTGTAG
ACeCA-LF	This work	TACCTCCTGTGAAGCTTGCTC
ACeCA-LB	This work	TTTAAAAGCATTGGGACCAGCGG
B-PR-F3	[1]	AAAGATAGGGGGGCAACT
B-PR-B3	[1]	GTTGACAGGTGTAGGTCCTA
B-PR-FIP	[1]	GGTTTCCATCTTCCTGGCAAATTTTTTCTCTATTAGATACAGGAGCAGA
B-PR-BIP	[1]	TGATAGGGGGAATTGGAGGTTTTTTTCCTATAGCTTTATGTCCACAGA
B-PR-LF	[1]	TTCCTATAGCTTTATGTCCACAGA
B-PR-LB	[1]	TATCAAAGTAAGACAGTA
A-PR-F3	This work	AARARTAGGRGGACAGCT
C-PR-F3	This work	AAAAGTAGGGGGCCAGRT
F-PR-F3	This work	AAAAGTAGGGGGACAGCT
AC-PR-F3b	This work	CACTCTTTGGCAACGACC
AC-PR-B3	This work	ATGTTGACAGGTGTAGGYCC
AC-PR-FIP	This work	GGTTTCCATCTTCCTGGCAAATTTTTTCTCTATTAGAYACAGGAGCAGA
AC-PR-BIPa	This work	TGATAGGRGGAATTGGAGGTTTTTTTCCTATAGCYTTWTKTCCACARA
AC-PR-BIPb	This work	TGATAGGRGGAATTGGAGGTTTTTTTGCYTTWTKTCCACARATTTCTA
AC-PR-LF	This work	TATDTCTTCTAATACTGTATCA
AC-PR-LB	This work	ATCAAAGTAAGACARTAT
B-RT-F3	[2]	AGTTCCCTTAGATAAAGACTT
B-RT-B3	[2]	CCTACATACAAATCATCCATGT
B-RT-FIP	[2]	GTGGAAGCACATTGTACTGATATCTTTTTGGAAGTATACTGCATTTACCAT
B-RT-BIP	[2]	GGAAAGGATCACCAGCAATATTCCTCTGGATTTTGTTTTCTAAAAGGC
B-RT-LF	[2]	GGTGTCTCATTGTTTATACTA
B-RT-LB	[2]	GCATGACAAAAATCTTAGA
ACeIN-F3	This work	TATTTGGAAAGGACCAGC
ACeIN-F3_b	This work	CGGGTTTATTACAGRGACAGCA
ACeIN-F3_c	This work	CCTATTTGGAAAGGACCAGC
ACeIN-F3_cL	This work	CCTATTTGGAAAGG+ACCAGC
ACeIN-B3a	This work	TCTTTGAAAYATACATATGRTG
ACeIN-B3a_L	This work	TCTTT+GAAAYATACATATGRTG
ACeIN-B3b	This work	AACATACATATGRTGYTTTACTA
ACeIN-B3bL	This work	AACA+TACATAT+GRTGYTTTACTA
ACeIN-FIPa	This work	CTTGGTACTACCTTTATGTCACTAAAGCTCCTCTGGAAAGGTG
ACeIN-FIPa_T	This work	CTTGGTACTACCTTTATGTCACTATTTTAAGCTCCTCTGGAAAGGTG
ACeIN-FIPb	This work	CTTGGCACTACTTTTATGTCACTAAAGCTCCTCTGGAAAGGTG
ACeIN-FIPb_T	This work	CTTGGCACTACTTTTATGTCACTATTTTAAGCTCCTCTGGAAAGGTG
ACeIN-FIPe	This work	CTTGGTACTACYTTTATGTCACTAAARCTACTCTGGAAAGGTG
ACeIN-FIPe_T	This work	CTTGGTACTACYTTTATGTCACTATTTTAARCTACTCTGGAAAGGTG
ACeIN-FIPe_L	This work	CTTGGTACTACYTTTA+TGT+CACTAAARC+TACTCT+GGAAAGGTG
ACeIN-FIPf	This work	CTTGGCACTACYTTTATGTCACTAAARCTYCTCTGGAAAGGTG
ACeIN-FIPf_T	This work	CTTGGCACTACYTTTATGTCACTATTTTAARCTYCTCTGGAAAGGTG

ACeIN-FIPf_L	This work	CTTGGCACTACYTTTATGTCACTAAARCTYCTCT+GGAAAGGTG
ACeIN-FIPg	This work	CTYCTTGGTACTACCTTTATGTCATACTCTGGAAAGGTGAAGG
ACeIN-FIPg_T	This work	CTYCTTGGTACTACCTTTATGTCATTTTTACTCTGGAAAGGTG
ACeIN-FIPh	This work	CTTCTTGGCACTACTTTTATGTCATYCTCTGGAAAGGTGAAGG
ACeIN-FIPh_T	This work	CTTCTTGGCACTACTTTTATGTCATTTTTYCTCTGGAAAGGTG
ACeIN-FIPi	This work	GGYACTACYTTTATGTCACTATTRTCCCTATTTGGAAAGGACCAGC
ACeIN-FIPi_T	This work	GGYACTACYTTTATGTCACTATTRTCTTTTCCTATTTGGAAAGGACCAGC
ACeIN-FIPj	This work	CTTGGTACTACCTTTATGTCACTAAAACTACTCTGGAAAGGTG
ACeIN-FIPj_T	This work	CTTGGTACTACCTTTATGTCACTATTTTAAACTACTCTGGAAAGGTG
ACeIN-FIPk	This work	CTTGGCACTACTTTTATGTCACTAAAGCTYCTCTGGAAAGGTG
ACeIN-FIPk_T	This work	CTTGGCACTACTTTTATGTCACTATTTTAAGCTYCTCTGGAAAGGTG
ACeIN-BIP	This work	GGAYTATGGAAAACAGATGGCAGCCATGTTCTAATCYTCATCCTG
ACeIN-BIP_T	This work	GGAYTATGGAAAACAGATGGCAGTTTTCCATGTTCTAATCYTCATCCTG
ACeIN-BIP_LT	This work	GGAYTATGGAAAA+CAGATGGCAGTTTTCCATGTTCTAA+TCYTCATCCTG
ACeIN-LF	This work	TCTTGTATTACTACTGCCCCTT
ACeIN-LF_b	This work	CTATTGTCTTGTATTACTACTGC
ACeIN-LF_c	This work	CTACTGCCCCTTCACCTTTCCA
ACeIN-LB	This work	GTGATGATTGTGTGGCARGTAG
F-IN-F3	This work	AGTTTGGAAAGGACCAGC
F-IN-B3a	This work	TCTTTGAAACATGCATATGGTA
F-IN-B3b	This work	AACATACATATGGTATTTTACTA
F-IN-FIP	This work	CTTGGTACTACCTTTATTTCACTAAAGCTACTCTGGAAAGGTG
F-IN-BIP	This work	GGATTATGGAAAACAGATGGCAGCCATGTGTTAATCCTCATCCTG
F-IN-LF	This work	CTTGTATGACTACTGCCCCTT
F-IN-LB	This work	GTGATGATTGTGTGGCAGGTAG

Note: “+” in sequences indicates that the following base is an LNA base.

References:

1. Curtis KA, Rudolph DL, Owen SM, Rapid detection of HIV-1 by reverse-transcription, loop-mediated isothermal amplification (RT-LAMP), J Virol Methods. 2008, 151(2): 264-70.
2. Curtis KA, Rudolph DL, Nejad I, Singleton J, Beddoe A, Weigl B, LaBarre P, Owen SM, Isothermal amplification using a chemical heating device for point-of-care detection of HIV-1. PLoS One. 2012;7(2):e31432.
