# Supplementary material for: A Reverse Transcription Loop-Mediated Isothermal Amplification Assay Optimized to Detect Multiple HIV Subtypes
Source: PLoS One. 2015 Feb 12;10(2):e0117852. doi: 10.1371/journal.pone.0117852 (PMC4326360; doi:10.1371/journal.pone.0117852)
Supplement: S2 Table — This table catalogs the primers used in each primer set. (PDF) [file pone.0117852.s003.pdf]

Table S2. HIV RT-LAMP primer sets tested

Primer set	Primers set name	Sources	Primers*
1	B-CA	[1]	B-CA-F3, B-CA-B3, B-CA-FIP, B-CA-BIP, B-CA-LF, B-CA-LB
2	B-PR	[1]	B-PR-F3, B-PR-B3, B-PR-FIP, B-PR-BIP, B-PR-LF, B-PR-LB
3	B-RT	[2]	B-RT-F3, B-RT-B3, B-RT-FIP, B-RT-BIP, B-RT-LF, B-RT-LB
4	B-CA + B-PR	This work	B-CA-F3, B-CA-B3, B-CA-FIP, B-CA-BIP, B-CA-LF, B-CA-LB, B-PR-F3, B-PR-B3, B-PR-FIP, B-PR-BIP, B-PR-LF, B-PR-LB
5	B-PR +B-RT	This work	B-PR-F3, B-PR-B3, B-PR-FIP, B-PR-BIP, B-PR-LF, B-PR-LB, B-RT-F3, B-RT-B3, B-RT-FIP, B-RT-BIP, B-RT-LF, B-RT-LB
6	ACeIN-1	This work	ACeIN-F3, ACeIN-B3a, ACeIN-FIPa, ACeIN-FIPb, ACeIN-BIP, ACeIN-LF, ACeIN-LB
7	ACeIN-2	This work	ACeIN-F3, ACeIN-B3b, ACeIN-FIPa, ACeIN-FIPb, ACeIN-BIP, ACeIN-LF, ACeIN-LB
8	ACeIN-3	This work	ACeIN-F3, ACeIN-B3a, ACeIN-B3b, ACeIN-FIPa, ACeIN-FIPb, ACeIN-BIP, ACeIN-LF, , ACeIN-LB
9	ACeCA	This work	ACeCA-F3, ACeCA-B3, ACeCA-FIP, ACeCA-BIP, ACeCA-LF, ACeCA-LB
10	ACeIN-4	This work	ACeIN-F3, ACeIN-B3a, ACeIN-B3b, ACeIN-FIPa, ACeIN-FIPb, ACeIN-BIP_T, ACeIN-LF, ACeIN-LB
11	ACeIN-5	This work	ACeIN-F3, ACeIN-B3a, ACeIN-B3b, ACeIN-FIPa_T, ACeIN-FIPb_T, ACeIN-BIP, ACeIN-LF, ACeIN-LB
12	ACeIN-6	This work	ACeIN-F3, ACeIN-B3a, ACeIN-B3b, ACeIN-FIPa_T, ACeIN-FIPb_T, ACeIN-BIP_T, ACeIN-LF, ACeIN-LB
13	ACeIN-7	This work	ACeIN-F3, ACeIN-B3a, ACeIN-B3b, ACeIN-FIPe, ACeIN-FIPf, ACeIN-BIP, ACeIN-LF, ACeIN-LB
14	ACeIN-8	This work	ACeIN-F3, ACeIN-B3a, ACeIN-B3b, ACeIN-FIPe, ACeIN-FIPf, ACeIN-BIP_T, ACeIN-LF, ACeIN-LB
15	ACeIN-9	This work	ACeIN-F3, ACeIN-B3a, ACeIN-B3b, ACeIN-FIPe_T, ACeIN-FIPf_T, ACeIN-BIP, ACeIN-LF, ACeIN-LB
16	ACeIN-10	This work	ACeIN-F3, ACeIN-B3a, ACeIN-B3b, ACeIN-FIPe_T, ACeIN-FIPf_T, ACeIN-BIP_T, ACeIN-LF, ACeIN-LB
17	ACeIN-11	This work	ACeIN-F3, ACeIN-B3a, ACeIN-B3b, ACeIN-FIPg, ACeIN-FIPh, ACeIN-BIP, ACeIN-LF_b, ACeIN-LB
18	ACeIN-12	This work	ACeIN-F3, ACeIN-B3a, ACeIN-B3b, ACeIN-FIPg, ACeIN-FIPh, ACeIN-BIP_T, ACeIN-LF_b, ACeIN-LB
19	ACeIN-13	This work	ACeIN-F3, ACeIN-B3a, ACeIN-B3b, ACeIN-FIPg_T, ACeIN-FIPh_T, ACeIN-BIP, ACeIN-LF_b, ACeIN-LB
20	ACeIN-14	This work	ACeIN-F3, ACeIN-B3a, ACeIN-B3b, ACeIN-FIPg_T, ACeIN-FIPh_T, ACeIN-BIP_T, ACeIN-LF_b, ACeIN-LB
21	ACeIN-15	This work	ACeIN-F3_b, ACeIN-B3a, ACeIN-B3b, ACeIN-FIPi, ACeIN-BIP, ACeIN-LF_c, ACeIN-LB
22	ACeIN-16	This work	ACeIN-F3_b, ACeIN-B3a, ACeIN-B3b, ACeIN-FIPi, ACeIN-BIP_T, ACeIN-LF_c, ACeIN-LB
23	ACeIN-17	This work	ACeIN-F3, ACeIN-B3a, ACeIN-B3b, ACeIN-FIPj, ACeIN-FIPk, ACeIN-BIP, ACeIN-LF, ACeIN-LB
24	ACeIN-18	This work	ACeIN-F3, ACeIN-B3a, ACeIN-B3b, ACeIN-FIPj, ACeIN-FIPk, ACeIN-BIP_T, ACeIN-LF, ACeIN-LB
25	ACeIN-19	This work	ACeIN-F3_b, ACeIN-B3a, ACeIN-B3b, ACeIN-FIPi_T, ACeIN-BIP, ACeIN-LF_c, ACeIN-LB
26	ACeIN-20	This work	ACeIN-F3_b, ACeIN-B3a, ACeIN-B3b, ACeIN-FIPi_T, ACeIN-BIP_T, ACeIN-LF_c, ACeIN-LB
27	ACeIN-21	This work	ACeIN-F3, ACeIN-B3a, ACeIN-B3b, ACeIN-FIPj_T, ACeIN-FIPk_T, ACeIN-BIP, ACeIN-LF, ACeIN-LB
28	ACeIN-22	This work	ACeIN-F3, ACeIN-B3a, ACeIN-B3b, ACeIN-FIPj_T, ACeIN-FIPk_T,

			ACeIN-BIP_T, ACeIN-LF, ACeIN-LB
29	ACeIN-23	This work	ACeIN-F3, ACeIN-F3_c, ACeIN-B3a, ACeIN-B3b, ACeIN-FIPa, ACeIN-FIPb, ACeIN-FIPe, ACeIN-FIPf, ACeIN-FIPj, ACeIN-FIPk, ACeIN-BIP, ACeIN-BIP_T, ACeIN-LF, ACeIN-LB
30	ACeIN-23+B-PR	This work	ACeIN-F3, ACeIN-F3_c, ACeIN-B3a, ACeIN-B3b, ACeIN-FIPa, ACeIN-FIPb, ACeIN-FIPe, ACeIN-FIPf, ACeIN-FIPj, ACeIN-FIPk, ACeIN-BIP, ACeIN-BIP_T, ACeIN-LF, ACeIN-LB, B-PR-F3, B-PR-B3, B-PR-FIP, B-PR-BIP, B-PR-LF, B-PR-LB
31	ACeIN-24	This work	ACeIN-F3_cL, ACeIN-B3a_L, ACeIN-B3b_L, ACeIN-FIPe, ACeIN-FIPf, ACeIN-BIP_LT, ACeIN-LF, ACeIN-LB
32	ACeIN-25	This work	ACeIN-F3_c, ACeIN-B3a, ACeIN-B3b, ACeIN-FIPa, ACeIN-FIPb, ACeIN-BIP, ACeIN-LF, ACeIN-LB
33	ACeIN-26	This work	ACeIN-F3_c, ACeIN-B3a, ACeIN-B3b, ACeIN-FIPe, ACeIN-FIPf, ACeIN-BIP, ACeIN-LF, ACeIN-LB
34	ACeIN-27	This work	ACeIN-F3_c, ACeIN-B3a, ACeIN-B3b, ACeIN-FIPe, ACeIN-FIPf, ACeIN-BIP_T, ACeIN-LF, ACeIN-LB
35	ACeIN-28	This work	ACeIN-F3_c, ACeIN-B3a, ACeIN-B3b, ACeIN-FIPj, ACeIN-FIPk, ACeIN-BIP, ACeIN-LF, ACeIN-LB
36	ACeIN-29	This work	ACeIN-F3_c, ACeIN-B3a, ACeIN-B3b, ACeIN-FIPj, ACeIN-FIPk, ACeIN-BIP_T, ACeIN-LF, ACeIN-LB
37	ACeIN-30	This work	ACeIN-F3_c, ACeIN-B3a, ACeIN-B3b, ACeIN-FIPe_L, ACeIN-FIPf_L, ACeIN-BIP, ACeIN-LF, ACeIN-LB
38	ACeIN-31	This work	ACeIN-F3_c, ACeIN-B3a, ACeIN-B3b, ACeIN-FIPe_L, ACeIN-FIPf_L, ACeIN-BIP_LT, ACeIN-LF, ACeIN-LB
39	ACeIN-26+B-PR	This work	ACeIN-F3_c, ACeIN-B3a, ACeIN-B3b, ACeIN-FIPe, ACeIN-FIPf, ACeIN-BIP, ACeIN-LF, ACeIN-LB, B-PR-F3, B-PR-B3, B-PR-FIP, B-PR-BIP, B-PR-LF, B-PR-LB
40	ACeIN-30+B-PR	This work	ACeIN-F3_c, ACeIN-B3a, ACeIN-B3b, ACeIN-FIPe_L, ACeIN-FIPf_Lm ACeIN-BIP, ACeIN-LF, ACeIN-LB, B-PR-F3, B-PR-B3, B-PR-FIP, B-PR-BIP, B-PR-LF, B-PR-LB
41	ACeIN-28+B-PR	This work	ACeIN-F3_c, ACeIN-B3a, ACeIN-B3b, ACeIN-FIPj, ACeIN-FIPk, ACeIN-BIP, ACeIN-LF, ACeIN-LB, B-PR-F3, B-PR-B3, B-PR-FIP, B-PR-BIP, B-PR-LF, B-PR-LB
42	ACeIN-32	This work	ACeIN-F3_cL, ACeIN-B3a, ACeIN-B3b, ACeIN-FIPe, ACeIN-FIPf ACeIN-BIP, ACeIN-LF, ACeIN-LB
43	ACeIN-33	This work	ACeIN-F3_c, ACeIN-B3a_L, ACeIN-B3b_L, ACeIN-FIPe, ACeIN-FIPf, ACeIN-BIP, ACeIN-LF, ACeIN-LB
44	ACeIN-34	This work	ACeIN-F3_cL, ACeIN-B3a_L, ACeIN-B3b_L, ACeIN-FIPe, ACeIN-FIPf, ACeIN-BIP, ACeIN-LF, ACeIN-LB
45	ACeIN-26+ACePR	This work	ACeIN-F3_c, ACeIN-B3a, ACeIN-B3b, ACeIN-FIPe, ACeIN-FIPf, ACeIN-BIP, ACeIN-LF, ACeIN-LB, A-PR-F3, B-PR-F3, C-PR-F3 ,F-PR-F3, AC-PR-F3b, AC-PR-B3, AC-PR-FIP, AC-PR-BIPa, AC-PR-BIPb, AC-PR-LF, AC-PR-LB
46	ACeIN-26+F-IN	This work	ACeIN-F3_c, ACeIN-B3a, ACeIN-B3b, ACeIN-FIPe, ACeIN-FIPf, ACeIN-BIP, ACeIN-LF, ACeIN-LB, F-IN-F3, F-IN-B3a, F-IN-B3b, F-IN-FIP, F-IN-BIP, F-IN-LF, F-IN-LB
47	ACeIN-26+F-IN+ACePR	This work	ACeIN-F3_c, ACeIN-B3a, ACeIN-B3b, ACeIN-FIPe, ACeIN-FIPf, ACeIN-BIP, ACeIN-LF, ACeIN-LB, F-IN-F3, F-IN-B3a, F-IN-B3b, F-IN-FIP, F-IN-BIP, F-IN-LF, F-IN-LB, A-PR-F3, B-PR-F3, C-PR-F3, F-PR-F3, AC-PR-F3b, AC-PR-B3, AC-PR-FIP, AC-PR-BIPa, AC-PR-BIPb, AC-PR-LF, AC-PR-LB

References:

1. Curtis KA, Rudolph DL, Owen SM, Rapid detection of HIV-1 by reverse-transcription, loop-mediated isothermal amplification (RT-LAMP), J Virol Methods. 2008, 151(2): 264-70.
2. Curtis KA, Rudolph DL, Nejad I, Singleton J, Beddoe A, Weigl B, LaBarre P, Owen SM, Isothermal amplification using a chemical heating device for point-of-care detection of HIV-1. PLoS One. 2012;7(2):e31432.
