# Supplementary material for: A Reverse Transcription Loop-Mediated Isothermal Amplification Assay Optimized to Detect Multiple HIV Subtypes
Source: PLoS One. 2015 Feb 12;10(2):e0117852. doi: 10.1371/journal.pone.0117852 (PMC4326360; doi:10.1371/journal.pone.0117852)
Supplement: S3 Table — Reactions contained 5000 copies of HIV-1 RNA templates from the subtypes listed at the tops of the columns. The threshold time (Tt) is defined as the reaction time that elapses until the fluorescent signal increases 10% of maximum fluorescence intensity (Imax) achieved in RNA-containing reactions. (PDF) [file pone.0117852.s004.pdf]

**Table S3.** Average threshold times (5000 copies).

"-" no test was performed. Threshold time of 61 min indicates lack of detectable signal during the experiment

| Clade | | A-UG | B | C | D | F | G | Average |
|---|---|---|---|---|---|---|---|---|
| Primer set | Primers set name | $T_t$ (min) | $T_t$ (min) | $T_t$ (min) | $T_t$ (min) | $T_t$ (min) | $T_t$ (min) | $T_t$ (min) |
| 1 | B-CA | – | 12.5 | 54.77 | 13.3 | 59.34 | – | 34.98 |
| 2 | B-PR | – | 16.07 | 61 | 15.38 | 51.85 | – | 36.08 |
| 3 | B-RT | – | 8.82 | 52.88 | 19.86 | 56.48 | – | 34.51 |
| 4 | B-CA + B-PR | – | 13.16 | 24.15 | 14.14 | 61 | – | 28.11 |
| 5 | B-PR +B-RT | – | 8.46 | 50.36 | 18.17 | 48.91 | – | 31.47 |
| 6 | ACeIN-1 | – | 10.91 | 24.45 | 51.63 | 53.99 | – | 35.25 |
| 7 | ACeIN-2 | – | 10.16 | 26.86 | 40.61 | 52.34 | – | 32.49 |
| 8 | ACeIN-3 | 14.37 | 9.12 | 22.43 | 25.17 | 50.52 | 14.43 | 22.67 |
| 9 | ACeCA | – | 32.34 | 61 | 61 | 61 | – | 53.84 |
| 10 | ACeIN-4 | – | 61 | 61 | 61 | 61 | – | 61 |
| 11 | ACeIN-5 | – | 55 | 61 | 61 | 61 | – | 59.5 |
| 12 | ACeIN-6 | – | 53.12 | 61 | 61 | 61 | – | 59.03 |
| 13 | ACeIN-7 | 14.09 | 9.04 | 14 | 21.3 | 40.81 | 19.08 | 19.72 |
| 14 | ACeIN-8 | 16.66 | 9.86 | 11.89 | 21.19 | 48.82 | 17.39 | 20.97 |
| 15 | ACeIN-9 | – | 34.35 | 61 | 61 | 61 | – | 54.34 |
| 16 | ACeIN-10 | – | 53.87 | 61 | 61 | 61 | – | 59.22 |
| 17 | ACeIN-11 | – | 16.07 | 25.36 | 61 | 51.47 | – | 38.47 |
| 18 | ACeIN-12 | – | 15.94 | 24.72 | 46.73 | 33.02 | – | 30.1 |
| 19 | ACeIN-13 | – | 34.81 | 45.74 | 61 | 61 | – | 50.64 |
| 20 | ACeIN-14 | – | 39.54 | 61 | 61 | 61 | – | 55.63 |

| | | | | | | | | |
|---|---|---|---|---|---|---|---|---|
| 21 | ACeIN-15 | – | 19.54 | 29.3 | 56.99 | 61 | – | 41.71 |
| 22 | ACeIN-16 | – | 26.45 | 31.12 | 41.26 | 61 | – | 39.96 |
| 23 | ACeIN-17 | 15.31 | 9.41 | 13.71 | 27.49 | 61 | 13.47 | 23.4 |
| 24 | ACeIN-18 | – | 13.39 | 17 | 25.26 | 43.91 | – | 24.89 |
| 25 | ACeIN-19 | – | 13.08 | 17.29 | 42.85 | 61 | – | 33.55 |
| 26 | ACeIN-20 | – | 15.38 | 20.55 | 61 | 61 | – | 39.48 |
| 27 | ACeIN-21 | – | 47.2 | 52.82 | 61 | 61 | – | 55.51 |
| 28 | ACeIN-22 | – | 46.73 | 59.34 | 61 | 61 | – | 57.02 |
| 29 | ACeIN-23 | | 10.82 | 16.13 | 24.46 | 61 | | 28.1 |
| 30 | ACeIN-23+B-PR | – | 10.45 | 17.14 | 17.62 | 33.89 | – | 19.77 |
| 31 | ACeIN-24 | – | 12.21 | 19.89 | 41.88 | 41.74 | – | 28.93 |
| 32 | ACeIN-25 | – | 8.91 | 14.02 | 14.41 | 46.49 | – | 20.96 |
| 33 | ACeIN-26 | 15.95 | 9.88 | 14.46 | 20.79 | 34.69 | 18.89 | 19.11 |
| 34 | ACeIN-27 | – | 12.55 | 37.7 | 36.48 | 61 | – | 36.93 |
| 35 | ACeIN-28 | 10.69 | 9.4 | 15.99 | 33.39 | 34.83 | 16.57 | 20.15 |
| 36 | ACeIN-29 | 14.02 | 10.68 | 14.87 | 26.12 | 31.39 | 35.95 | 22.17 |
| 37 | ACeIN-30 | 16.69 | 11.33 | 20.64 | 29.01 | 43.2 | 24.25 | 24.19 |
| 38 | ACeIN-31 | 23.25 | 15.02 | 21.67 | 61 | 46.37 | 24.62 | 31.99 |
| 39 | ACeIN-26+B-PR | 16.1 | 9.05 | 14.73 | 14.8 | 51.26 | 14.05 | 20 |
| 40 | ACeIN-30+B-PR | 29.95 | 10.38 | 28.49 | 20.33 | 42.46 | 23.09 | 25.78 |
| 41 | ACeIN-28+B-PR | 11.44 | 9.93 | 33.62 | 21.75 | 46.72 | 13.41 | 22.81 |
| 42 | ACeIN-32 | 16.16 | 10.39 | 15.98 | 29.17 | 51.34 | 17.71 | 23.46 |
| 43 | ACeIN-33 | 16.27 | 10.48 | 15.81 | 22.75 | 50.48 | 16.79 | 22.1 |
| 44 | ACeIN-34 | 15.9 | 10.22 | 14.35 | 22.63 | 51.99 | 27.15 | 23.71 |
| 45 | ACeIN-26+ACePR | 21.66 | 12.16 | 23.47 | 39.13 | 50.33 | 26.09 | 28.81 |
| 46 | ACeIN-26+F-IN | 14.51 | 12.35 | 15.06 | 13.54 | 43.39 | 15.61 | 19.08 |
| 47 | ACeIN-26+F-IN+ACePR | 19.69 | 13.08 | 18.45 | 14.91 | 35.13 | 16.09 | 19.56 |
