# Supplementary material for: A Reverse Transcription Loop-Mediated Isothermal Amplification Assay Optimized to Detect Multiple HIV Subtypes
Source: PLoS One. 2015 Feb 12;10(2):e0117852. doi: 10.1371/journal.pone.0117852 (PMC4326360; doi:10.1371/journal.pone.0117852)
Supplement: S4 Table — This table documents the raw values for results of this study. (PDF) [file pone.0117852.s005.pdf]

Table S4. All threshold times generated in this study (5000 target copies)

Primer set	Primer sets name	Experiment	A-UG	B	C	D	F	G	Average
			T_t (min)	T_t (min)	T_t (min)	T_t (min)	T_t (min)	T_t (min)	T_t (min)
**1**	B-CA	1.1		13.29	48.54	13.31	52.68		
		1.2		11.9	61	13.49	61		
		1.3		12.47		11.86	61		
		1.4		12.41		14.46	61		
		1.5		12.45		13.4	61		
		**Average**		**12.5**	**54.77**	**13.3**	**59.34**		**34.98**
		STD		0.5	8.81	0.93	5.89		4.03
**2**	B-PR	2.1		15.88	61	15.91	55.54		
		2.2		16.35	61	16.93	61		
		2.3		15.83	61	15.93	61		
		2.4		16.65	61	14.47	61		
		2.5		15.66	61	13.67	20.69		
		**Average**		**16.07**	**61**	**15.38**	**51.85**		**36.08**
		STD		0.41	0	1.3	3.86		1.39
**3**	B-RT	3.1		9.18	61	12.6	41.78		
		3.2		8.88	48.97	19.88	61		
		3.3		8.9	61	21.95	61		
		3.4		8.64	36.63	26.25	61		
		3.5		8.52	56.79	18.64	57.61		
		**Average**		**8.82**	**52.88**	**19.86**	**56.48**		**34.51**
		STD		0.25	10.33	4.99	13.59		7.29
**4**	B-CA + B-PR	4.1		13.4	16.58	13.77	61		
		4.2		12.93	31.71	14.5	61		

Primer set	Primer sets name	Experiment	A-UG	B	C	D	F	G	Average
			T_t (min)	T_t (min)	T_t (min)	T_t (min)	T_t (min)	T_t (min)	T_t (min)
		**Average**		**13.16**	**24.15**	**14.14**	**61**		**28.11**
		STD		0.33	10.7	0.52	0		2.89
**5**	B-PR +B-RT	5.1		8.46	39.71	19.39	46.48		
		5.2		8.47	61	16.94	51.33		
		**Average**		**8.46**	**50.36**	**18.17**	**48.91**		**31.47**
		STD		0	15.05	1.73	3.43		5.05
**6**	ACelN-1	6.1		10.98	35.57	56.75	50.4		
		6.2		10.84	13.33	46.51	57.59		
		**Average**		**10.91**	**24.45**	**51.63**	**53.99**		**35.25**
		STD		0.1	15.73	7.24	5.08		7.04
**7**	ACelN-2	7.1		10.41	34.39	49.78	55.94		
		7.2		9.92	19.33	31.45	48.74		
		**Average**		**10.16**	**26.86**	**40.61**	**52.34**		**32.49**
		STD		0.34	10.65	12.96	5.09		7.26
**8**	ACelN-3	8.1	16.51	9.28	33.58	27.5	54.62	15.58	
		8.2	12.22	9.67	61	41.48	61	13.27	
		8.3		9.97	13.4	12.57	61		
		8.4		8.66	14.36	18.94	61		
		8.5		9.26	12.97	13.88	18.83		
		8.6		9.36	15.34	12.58	61		
		8.7		8.3	14.29	61	25.69		
		8.8		8.48	14.53	13.37	61		
		**Average**	**14.37**	**9.12**	**22.43**	**25.17**	**50.52**	**14.43**	**22.67**
		STD	3.03	0.59	17.02	17.64	17.67	1.64	9.6

Primer set	Primer sets name	Experiment	A-UG	B	C	D	F	G	Average
			T_t (min)	T_t (min)	T_t (min)	T_t (min)	T_t (min)	T_t (min)	T_t (min)
**9**	ACeCA	9.1		32.88	61	61	61		
		9.2		31.81	61	61	61		
		**Average**		**32.34**	**61**	**61**	**61**		**53.84**
		STD		0.75	0	0	0		0.19
**10**	ACeIN-4	10.1		61	61	61	61		
		10.2		61	61	61	61		
		**Average**		**61**	**61**	**61**	**61**		**61**
		STD		0	0	0	0		0
**11**	ACeIN-5	11.1		48.99	61	61	61		
		11.2		61	61	61	61		
		**Average**		**55**	**61**	**61**	**61**		**59.5**
		STD		8.49	0	0	0		2.12
**12**	ACeIN-6	12.1		54.75	61	61	61		
		12.2		51.48	61	61	61		
		**Average**		**53.12**	**61**	**61**	**61**		**59.03**
		STD		2.31	0	0	0		0.58
**13**	ACeIN-7	13.1	15.81	9.4	13.48	16.81	19.31	15.55	
		13.2	12.37	9.66	15.64	32.86	41.6	22.62	
		13.3		8.43	11.48	17.96	61		
		13.4		8.68	15.39	17.57	41.33		
		**Average**	**14.09**	**9.04**	**14**	**21.3**	**40.81**	**19.08**	**19.72**
		STD	2.44	0.5	1.68	6.69	14.75	5	5.18
**14**	ACeIN-8	14.1	20.37	10.54	12.47	16.8	21.8	12.96	
		14.2	12.95	9.94	10.71	14.68	61	21.83	

Primer set	Primer sets name	Experiment	A-UG	B	C	D	F	G	Average
			T_t (min)	T_t (min)	T_t (min)	T_t (min)	T_t (min)	T_t (min)	T_t (min)
		14.3		9.49	12.38	30.68	61		
		14.4		9.46	11.99	22.59	51.48		
		**Average**	**16.66**	**9.86**	**11.89**	**21.19**	**48.82**	**17.39**	**20.97**
		STD	5.24	0.44	0.71	6.2	16.08	6.27	5.82
**15**	ACeIN-9	15.1		35.85	61	61	61		
		15.2		32.85	61	61	61		
		**Average**		**34.35**	**61**	**61**	**61**		**54.34**
		STD		2.12	0	0	0		0.53
**16**	ACeIN-10	16.1		61	61	61	61		
		16.2		46.75	61	61	61		
		**Average**		**53.87**	**61**	**61**	**61**		**59.22**
		STD		10.08	0	0	0		2.52
**17**	ACeIN-11	17.1		16.43	26.34	61	41.93		
		17.2		15.71	24.39	61	61		
		**Average**		**16.07**	**25.36**	**61**	**51.47**		**38.47**
		STD		0.51	1.38	0	13.48		3.84
**18**	ACeIN-12	18.1		16.58	24.46	61	25.39		
		18.2		15.29	24.98	32.46	40.64		
		**Average**		**15.94**	**24.72**	**46.73**	**33.02**		**30.1**
		STD		0.91	0.37	20.18	10.78		8.06
**19**	ACeIN-13	19.1		33.65	48.68	61	61		
		19.2		35.97	42.79	61	61		
		**Average**		**34.81**	**45.74**	**61**	**61**		**50.64**
		STD		1.64	4.16	0	0		1.45

Primer set	Primer sets name	Experiment	A-UG	B	C	D	F	G	Average
			T_t (min)	T_t (min)	T_t (min)	T_t (min)	T_t (min)	T_t (min)	T_t (min)
**20**	ACeIN-14	20.1		37.59	61	61	61		
		20.2		41.49	61	61	61		
		**Average**		**39.54**	**61**	**61**	**61**		**55.63**
		STD		2.76	0	0	0		0.69
**21**	ACeIN-15	21.1		20.1	24.66	52.99	61		
		21.2		18.99	33.93	61	61		
		**Average**		**19.54**	**29.3**	**56.99**	**61**		**41.71**
		STD		0.79	6.56	5.67	0		3.25
**22**	ACeIN-16	22.1		25.97	30.71	30.99	61		
		22.2		26.94	31.53	51.53	61		
		**Average**		**26.45**	**31.12**	**41.26**	**61**		**39.96**
		STD		0.68	0.58	14.53	0		3.95
**23**	ACeIN-17	23.1	14.7	10.23	13.84	12.83	61	12.34	
		23.2	15.92	9.58	12.47	22.66	61	14.6	
		23.3		8.85	16.74	34.89	61		
		23.4		8.99	11.8	39.57	61		
		**Average**	**15.31**	**9.41**	**13.71**	**27.49**	**61**	**13.47**	**23.4**
		STD	0.87	0.55	1.9	10.48	0	1.6	2.56
**24**	ACeIN-18	24.1		14.35	14.49	30.99	26.83		
		24.2		12.43	19.51	19.52	61		
		**Average**		**13.39**	**17**	**25.26**	**43.91**		**24.89**
		STD		1.36	3.55	8.12	24.16		9.3
**25**	ACeIN-19	25.1		13.51	15.83	24.7	61		
		25.2		12.65	18.75	61	61		

Primer set	Primer sets name	Experiment	A-UG	B	C	D	F	G	Average
			T_t (min)	T_t (min)	T_t (min)	T_t (min)	T_t (min)	T_t (min)	T_t (min)
		**Average**		**13.08**	**17.29**	**42.85**	**61**		**33.55**
		STD		0.61	2.06	25.67	0		7.09
26	ACeIN-20	26.1		16.96	20.65	61	61		
		26.2		13.8	20.45	61	61		
		**Average**		**15.38**	**20.55**	**61**	**61**		**39.48**
		STD		2.23	0.14	0	0		0.59
27	ACeIN-21	27.1		49.96	61	61	61		
		27.2		44.45	44.64	61	61		
		**Average**		**47.2**	**52.82**	**61**	**61**		**55.51**
		STD		3.9	11.57	0	0		3.87
28	ACeIN-22	28.1		42.81	57.68	61	61		
		28.2		50.66	61	61	61		
		**Average**		**46.73**	**59.34**	**61**	**61**		**57.02**
		STD		5.55	2.35	0	0		1.98
29	ACeIN-23	29.1		11.69	13.99	29.43	61		
		29.2		9.95	18.28	19.49	61		
		**Average**		**10.82**	**16.13**	**24.46**	**61**		**28.1**
		STD		1.23	3.04	7.03	0		2.83
30	AceIN-23+B-PR	30.1		10.93	17.5	18.4	29.55		
		30.2		9.97	16.78	16.84	38.22		
		**Average**		**10.45**	**17.14**	**17.62**	**33.89**		**19.77**
		STD		0.68	0.51	1.1	6.13		2.1
31	ACeIN-24	31.1		12.54	19.81	40.95	61		
		31.2		11.88	19.97	42.81	22.47		

Primer set	Primer sets name	Experiment	A-UG	B	C	D	F	G	Average
			T_t (min)	T_t (min)	T_t (min)	T_t (min)	T_t (min)	T_t (min)	T_t (min)
		**Average**		**12.21**	**19.89**	**41.88**	**41.74**		**28.93**
		STD		0.46	0.12	1.32	27.24		7.29
**32**	ACeIN-25	32.1		9.16	12.53	13.92	31.99		
		32.2		8.65	15.51	14.91	61		
		**Average**		**8.91**	**14.02**	**14.41**	**46.49**		**20.96**
		STD		0.36	2.11	0.7	20.51		5.92
**33**	ACeIN-26	33.1	13.52	9.96	15.25	18.75	46.36	13.99	
		33.2	14.25	10.99	13.32	18.36	19.79	18.72	
		33.3	17.41	9.4	15.59	24.42	33.7	33.9	
		33.4	15.59	8.96	14.68	14.82	22.24	12.36	
		33.5	20.4	10.22	13.6	29.6	61	14.81	
		33.6	14.55	11.22	13.96	20.81	61	19.56	
		33.7		9.64	11.6	18.62	15.79		
		33.8		8.63	17.66	20.93	17.67		
		**Average**	**15.95**	**9.88**	**14.46**	**20.79**	**34.69**	**18.89**	**19.11**
		STD	2.56	0.91	1.8	4.49	19.07	7.86	6.12
**34**	ACeIN-27	34.1		12.51	61	35.82	61		
		34.2		12.59	14.4	37.14	61		
		**Average**		**12.55**	**37.7**	**36.48**	**61**		**36.93**
		STD		0.06	32.95	0.94	0		8.49
**35**	ACeIN-28	35.1	10.43	9.28	10.79	28.57	34.4	16.21	
		35.2	10.95	9.54	17.33	30.61	13.97	16.93	
		35.3		9.5	18.47	13.39	61		
		35.4		9.26	17.37	61	29.95		

Primer set	Primer sets name	Experiment	A-UG	B	C	D	F	G	Average
			T_t (min)	T_t (min)	T_t (min)	T_t (min)	T_t (min)	T_t (min)	T_t (min)
		**Average**	**10.69**	**9.4**	**15.99**	**33.39**	**34.83**	**16.57**	**20.15**
		STD	0.37	0.13	3.04	17.27	16.91	0.51	6.37
**36**	ACeIN-29	36.1	12.72	8.96	17.67	20.4	61	61	
		36.2	15.32	10.92	15.61	21.74	24.46	10.89	
		36.3		12.28	12.37	34.77	21.7		
		36.4		10.55	13.82	27.55	18.42		
		**Average**	**14.02**	**10.68**	**14.87**	**26.12**	**31.39**	**35.95**	**22.17**
		STD	1.84	1.18	1.98	5.67	17.23	35.43	10.56
**37**	ACeIN-30	37.1	15.42	11.9	23.39	34.57	61	19.92	
		37.2	17.97	10.76	17.88	23.46	25.41	28.58	
		**Average**	**16.69**	**11.33**	**20.64**	**29.01**	**43.2**	**24.25**	**24.19**
		STD	1.8	0.81	3.89	7.85	25.17	6.12	7.61
**38**	ACeIN-31	38.1	20.93	14.54	19.5	61	31.74	26.55	
		38.2	25.56	15.5	23.84	61	61	22.7	
		**Average**	**23.25**	**15.02**	**21.67**	**61**	**46.37**	**24.62**	**31.99**
		STD	3.27	0.68	3.06	0	20.69	2.73	5.07
**39**	ACeIN-26+B-PR	39.1	13.68	8.45	15.84	14.68	61	11.77	
		39.2	18.52	9.65	13.61	14.93	41.52	16.33	
		**Average**	**16.1**	**9.05**	**14.73**	**14.8**	**51.26**	**14.05**	**20**
		STD	3.42	0.85	1.58	0.18	13.77	3.23	3.84
**40**	ACeIN-30+B-PR	40.1	43.96	10.27	44.57	20.69	61	26.6	
		40.2	15.94	10.5	12.4	19.97	23.93	19.58	
		**Average**	**29.95**	**10.38**	**28.49**	**20.33**	**42.46**	**23.09**	**25.78**
		STD	19.81	0.16	22.75	0.51	26.22	4.96	12.4

Primer set	Primer sets name	Experiment	A-UG	B	C	D	F	G	Average
			T_t (min)	T_t (min)	T_t (min)	T_t (min)	T_t (min)	T_t (min)	T_t (min)
**41**	ACeIN-28+B-PR	41.1	12.49	10.39	54.62	18.65	61	12.93	
		41.2	10.4	9.46	12.63	24.85	32.44	13.88	
		**Average**	**11.44**	**9.93**	**33.62**	**21.75**	**46.72**	**13.41**	**22.81**
		STDE	1.48	0.66	29.7	4.39	20.19	0.68	9.51
**42**	ACeIN-32	42.1	12.8	9.94	16.35	19.75	61	15.8	
		42.2	15.38	10.68	14.89	46.73	61	18.6	
		42.3	17.86	10.51	17.86	24.72	22.38	17.96	
		42.4	18.61	10.45	14.84	25.47	61	18.47	
		**Average**	**16.16**	**10.39**	**15.98**	**29.17**	**51.34**	**17.71**	**23.46**
		STD	2.63	0.32	1.43	11.98	19.31	1.3	6.16
**43**	ACeIN-33	43.1	13.52	9.86	12.26	26.99	61	13.46	
		43.2	14.84	10.5	16.32	19.78	61	15.68	
		43.3	20.48	10.96	18.93	17.44	27.23	15.37	
		43.4	16.26	10.6	15.74	26.77	52.68	22.65	
		**Average**	**16.27**	**10.48**	**15.81**	**22.75**	**50.48**	**16.79**	**22.1**
		STD	3.02	0.46	2.74	4.87	15.99	4.03	5.18
**44**	ACeIN-34	44.1	15.88	10.33	13.68	16.45	24.95	19.51	
		44.2	15.82	9.47	14.57	31.59	61	14.7	
		44.3	15.47	10.6	13.44	15.6	61	61	
		44.4	16.43	10.47	15.7	26.88	61	13.4	
		**Average**	**15.9**	**10.22**	**14.35**	**22.63**	**51.99**	**27.15**	**23.71**
		STD	0.4	0.51	1.02	7.87	18.02	22.72	8.42
**45**	ACeIN-26+ACePR	45.1	21.81	12.8	14.53	46.97	39.66	17.29	
		45.2	21.52	11.53	32.41	31.29	61	34.88	

Primer set	Primer sets name	Experiment	A-UG	B	C	D	F	G	Average
			T_t (min)	T_t (min)	T_t (min)	T_t (min)	T_t (min)	T_t (min)	T_t (min)
		**Average**	**21.66**	**12.16**	**23.47**	**39.13**	**50.33**	**26.09**	**28.81**
		STD	0.2	0.9	12.64	11.08	15.09	12.44	8.72
**46**	ACeIN-26+F-IN	46.1	14.46	12.51	14.55	14.44	61	16.97	
		46.2	14.57	12.18	15.58	12.65	25.78	14.25	
		**Average**	**14.51**	**12.35**	**15.06**	**13.54**	**43.39**	**15.61**	**19.08**
		STD	0.08	0.23	0.73	1.26	24.91	1.93	4.86
**47**	ACeIN-26+F-IN+ACePR	47.1	22.62	14.35	19.98	15.94	44.97	13.74	
		47.2	16.76	11.81	16.92	13.89	25.29	18.44	
		**Average**	**19.69**	**13.08**	**18.45**	**14.91**	**35.13**	**16.09**	**19.56**
		STD	4.15	1.79	2.16	1.45	13.92	3.32	4.47

Samples consist of 5000 copies per reaction of HIV-1 templates. The threshold time (T_t) is defined as the reaction time that elapses until the florescent signal increases to 10% of maximum fluorescence intensity (I_{max}) above the baseline level. For cases where there was no amplification, we designate 61 min as T_t. Lack of entry indicates that no experiment was carried out.
